# Transcriptomic Insights into the Protective Effects of Apigenin and Sodium Butyrate on Jejunal Oxidative Stress in Ducks

**DOI:** 10.3390/vetsci12070655

**Published:** 2025-07-11

**Authors:** Ning Zhou, Hanxue Sun, Yong Tian, Heng Zhang, Xuemei Xian, Hui Yu, Lingyan Zhao, Yong Chen, Mingkun Sun, Yiqian Zhang, Ting Meng, Lizhi Lu

**Affiliations:** 1 College of Pet Sciences, Jiangsu Agri-Animal Husbandry Vocational College, Taizhou 225300, China; 2022010534@jsahvc.edu.cn (N.Z.); zhrealm@163.com (H.Z.); 2024010599@jsahvc.edu.cn (X.X.); 15849123986@163.com (M.S.); zyq1027219294@163.com (Y.Z.); 2 Zhejiang Provincial Key Laboratory of Livestock and Poultry Biotech Breeding, Key Laboratory of Livestock and Poultry Resources (Poultry) Evaluation and Utilization, Ministry of Agriculture and Rural Affairs, State Key Laboratory for Quality and Safety of Agro-Products, Institute of Animal Science & Veterinary, Zhejiang Academy of Agricultural Sciences, Hangzhou 310021, China; sunhx@zaas.ac.cn (H.S.); tyong@zaas.ac.cn (Y.T.); 3 College of Animal Science and Technology, Nanjing Agricultural University, Nanjing 210095, China; 4 Zhejiang Xinchang Agricultural Development Co., Ltd., Lishui 321400, China; 13346077806@163.com; 5 Zhejiang Provincial Center for Animal Disease Prevention and Control, Zhejiang Provincial Institute of Veterinary Drug and Feedstuff, Hangzhou 310021, China; zhaoly96@163.com (L.Z.); xiaoyong97121006@163.com (Y.C.)

**Keywords:** feed additive, diquat, Jinyun Ma ducks, jejunal morphology, oxidative injury

## Abstract

Apigenin and sodium butyrate have been reported to help alleviate oxidative stress. This study evaluated the jejunal transcriptomic responses in ducks receiving apigenin and sodium butyrate supplementation under oxidative stress. In total, 200 healthy 300-day-old female Jinyun Ma ducks (1.53 kg ± 0.15) were randomly divided into four groups, with five replicates per group. The groups were as follows: a control group (CON): ducks were fed a basal diet with sterile saline injection; a diquat-injection (DIQ) group: ducks were fed a basal diet with diquat injection; an apigenin plus diquat group (API): ducks were fed a basal diet containing apigenin (500 mg/kg) with diquat injection; and a sodium butyrate plus diquat group (SB): ducks were fed a basal diet containing sodium butyrate (500 mg/kg) with diquat injection. The injection dose of diquat is 8 mg/kg body weight. Analysis revealed that the dietary supplementation of apigenin and sodium butyrate reduced malondialdehyde (MDA) levels and increased total antioxidant capacity (T-AOC) (*p* < 0.05). Compared to the DIQ group, sodium butyrate supplementation during oxidative stress elevated jejunal villus height and villus height/crypt depth ratio in ducks (*p* < 0.05). The study identified that some candidate genes, including solute carrier family 4 member 3 (*SLC4A3*), ADAM metallopeptidase domain 12 (*ADAM12*), and B-cell lymphoma 2-associated-athanogene 3 (*BAG3*), were significantly upregulated, whereas claudin 23 (*CLDN23*) and glucose-6-phosphatase catalytic subunit 1 (*G6PC1*) were markedly downregulated in the API group in comparison with that in the DIQ group (*p* < 0.05). Collectively, our findings provide molecular evidence for the beneficial effects of apigenin and sodium butyrate against oxidative stress in the jejunum of ducks.

## Simple Summary

Under intensive industrial farming conditions, ducks are susceptible to oxidative stress. The small intestine is the dominant interface responsible for the exchange of substances and energy between organisms and their environment. Oxidative stress is thought to trigger rapid changes in the intestinal microbiota that can lead to pathological symptoms and even death. Apigenin and sodium butyrate have been reported to help alleviate oxidative stress; consequently, we administered these two additives to the diet of ducks under oxidative stress. Analysis revealed that dietary supplementation with apigenin and sodium butyrate alleviated diquat-induced impaired intestinal morphological injury, redox imbalance, and transcriptome level in the jejunum.

## 1. Introduction

The maintenance of duck health during intensive farming efforts has become a major research theme in livestock husbandry. Under intensive and industrial farming conditions, ducks are highly susceptible to a range of stressors, including oxidative, heat, and cage stress; of these, oxidative stress is the most prevalent [1]. Oxidative stress arises from disrupted homeostasis between reactive oxygen species (ROS) production and elimination [2]. Under these circumstances, notable changes occur in the levels of certain enzymes, including superoxide dismutase (SOD), glutathione peroxidase (GSH-PX), and catalase (CAT), along with changes in related biomarkers, including total antioxidant capacity (T-AOC) and malondialdehyde (MDA) [3].

The small intestine is the dominant interface responsible for the exchange of substances and energy between organisms and their environment. The small intestine is a dynamic organ involved in nutrient absorption, barrier function, and immune signaling to facilitate digestion and maintain energetic homeostasis [4,5]. However, oxidative stress can trigger rapid changes in the intestinal microbiota that can lead to pathological symptoms and even death [6]. The jejunum, a specific region of the small intestine, exerts functionality by absorbing nutrients and is, therefore, a key biological structure for the rapid growth of ducks [7]. Oxidative stress-induced apoptosis and mucosal barrier impairment in animal intestines are established in a previous study [8]. Therefore, understanding and addressing intestinal health issues related to oxidative stress in ducks, particularly under intensive farming environments, is crucial for livestock yield and product quality. Although these effects were reported in broilers, similar physiological responses may occur in ducks. There is a significant need to investigate antioxidant feed additives that could attenuate jejunal oxidative damage in ducks under intensive farming conditions.

Diquat is a redox circulator and produces superoxide anions and other redox products to induce oxidative stress [9]. Consequently, diquat is used to generate in vivo models of oxidative stress [10]. Other researchers have shown that plant-derived feed additives represent an effective strategy to enhance the health and performance of animals in intensive production systems [11,12]. Apigenin, a dietary flavonoid, occurs naturally in various fruits and vegetables and is known to exhibit both antioxidant and anti-apoptotic effects in a variety of systematic disorders [13,14,15,16]. Over recent years, researchers have shown that apigenin can exert beneficial effects on several diseases with good levels of biosafety, including neurodegenerative diseases and retinal degeneration [17,18].

Sodium butyrate, an inorganic salt of butyric acid, can be produced by intestinal microorganisms via the fermentation of dietary carbohydrates and fibers [19]. Sodium butyrate was shown to exhibit protective effects on the intestine and improve the integrity of the intestinal barrier [20]. Previous studies in poultry have demonstrated that sodium butyrate exerts beneficial effects on growth performance, intestinal morphology, intestinal immunity, intestinal microflora, and anti-oxidative capacities [21,22]. However, there is limited knowledge about the effects of sodium butyrate on duck intestinal health under oxidative stress.

Transcriptome sequencing is a comprehensive technique that can analyze the entire set of global transcripts at the tissue or whole organism levels and can, therefore, reveal the molecular mechanisms involved in certain biological processes, thus providing a key foundation for the investigation of gene regulation, which is able to influence traits to select candidate genes to improve the duck breeding industry [23,24]. Poultry subjected to oxidative stress often exhibits changes in gene expression; the genes involved in the regulation of oxidative balance include: *NRF2*, *GPx4*, *KEAP1*, *AHR*, *NQO1*, *NOX2*, and *SOD2* [25,26].

This work characterized the influence of apigenin and sodium butyrate on the jejunal anti-oxidative index and analyzed the mechanism by which apigenin and sodium butyrate can influence intestinal damage caused by oxidative stress in ducks. Our findings could provide a valuable foundation for the future prevention and treatment of oxidative stress, establishing a basis for the application of antioxidant feed additives.

## 2. Materials and Methods

### 2.1. Experimental Design and Animal Feeding

In total, 200 healthy female Jinyun Ma ducks (1.53 kg ± 0.15) were selected and randomly assigned to four treatment groups, each containing five replicates with 10 ducks per replicate. For consistency, 300-day-old female ducks were selected with similar body weights. The four treatment groups were as follows: (1) control group (CON): ducks were fed a basal diet with a sterile saline injection; (2) diquat group (DIQ): ducks were fed a basal diet and injected with diquat; (3) apigenin group (API): ducks were fed a basal diet containing apigenin (Staherb, Changsha, China, 500 mg/kg) with diquat injection; (4) sodium butyrate group (SB): ducks were fed a basal diet containing sodium butyrate (King Techina, Hangzhou, China, 500 mg/kg) with diquat injection. Dietary composition and nutrient profiles are given in Table 1 and follow the National Research Council (NRC, 1998) guidelines. The entire experimental period lasted 20 days (d) and was separated into a 10-day pre-feeding trial and a 10-day dietary intervention. Experimental ducks from the DIQ, RES, and API groups received an intraperitoneal injection of diquat at 8 mg/kg body weight [9]. The experimental ducks were reared in three-tier battery cages, with a cage size of 40 × 50 × 40 cm per replicate. Ducks were maintained at room temperature with controlled ventilation and humidity levels of 50–70% and were kept under standard lighting conditions (light/dark: 10 h/14 h) during the experimental period. Food and water were provided ad libitum. The research was performed at Jinyun Ma Duck Breeding Farm, Jinyun County, Lishui, Zhejiang Province. All protocols received approval from the Institutional Animal Care and Use Committee at the Jiangsu Agri-animal Husbandry Vocational College (Permit Number: JSAHVC-2023-10).

### 2.2. Sample Collection

A total of 20 ducks were randomly selected from each replicate and humanely sacrificed and dissected following overnight feed deprivation. Subsequently, the entire intestine was immediately removed. Two samples of jejunum tissue were collected after rinsing with ice-cold phosphate-buffered saline (PBS, Solarbio, Beijing, China). One sample was flushed gently to remove luminal chyme and then stored in liquid nitrogen to await the detection of antioxidant parameters and transcriptome analysis. The other sample was fixed in 4% paraformaldehyde (Biosharp, Shanghai, China) for histopathological analysis [8].

### 2.3. Detection of Oxidative Stress Markers in the Jejunum

Frozen jejunal tissue was pulverized in liquid nitrogen, homogenized with a tissue homogenizer (Bullet Blender, Next Advance, Inc., Troy, NY, USA), and then centrifuged at 3500× *g* for 10 min to acquire a supernatant, which was then stored at −20 °C to evaluate redox status indicators. Levels of malondialdehyde (MDA) and catalase (CAT), total antioxidative capacity (T-AOC), along with the activities of superoxide dismutase (SOD) and glutathione peroxidase (GSH-PX) in the jejunal mucosa were analyzed by commercial kits (Nanjing Jiancheng Bioengineering Institute, Nanjing, China) following the manufacturer’s protocols.

### 2.4. Jejunal Morphology Analysis

After dehydration and embedding, 5-μm sections of intestinal tissue were prepared and H&E-stained. Then, an optical microscope (Olympus BX5, Tokyo, Japan) and digital camera (Nikon, Tokyo, Japan) were employed to quantify villus height and crypt depth per sample, in accordance with our previous study [8]. The jejunal villus height and crypt depth were measured using Image-Pro Plus 6.0 (Bethesda, Rockville, MD, USA).

### 2.5. RNA Extraction, Library Preparation, and Sequencing

Total RNA was isolated from jejunal tissues with an RNA Kit (OMEGA Bio-Tek, Norcross, GA, USA) in accordance with the manufacturer’s instructions. RNA quantity was determined by a NanoDrop (Thermo Fisher, Waltham, MA, USA) while RNA quality was determined with an Agilent2100 Bioanalyzer (Agilent Technologies, Santa Clara, CA, USA). Samples with an RNA integrity number (RIN) ≥ 7 were used for cDNA library construction using a TruSeqTM RNA sample preparation Kit from Illumina (San Diego, CA, USA) in accordance with the manufacturer’s guidelines. mRNA was purified by poly-T oligo-attached magnetic beads and subjected to randomized fragmentation using fragmentation buffer. First-strand cDNA synthesis was performed using random hexamers as primers and the fragmented mRNA as a template, followed by second-strand synthesis and the addition of PCR buffer, DNA polymerase I, RNase H, and dNTPs; the cDNA was purified with AMPure XP beads. Further, we generated a cDNA library by PCR and sequenced paired-end libraries on the Illumina HiSeq 2500 platform (Illumina Inc., San Diego, CA, USA) to obtain 150 bp paired-end reads.

### 2.6. Quality Control, Quantification, and Differential Expression Analysis

The raw sequencing reads (Illumina) were quality-filtered by removing adapters, poly-N, and low-quality reads. Then, we calculated quality parameters. The filtered reads were then mapped to the duck reference genome and genomic annotations acquired from NCBI (http://www.ncbi.nlm.nih.gov/) URL (accessed on 25 November 2023) using Hisat2 (https://ccb.jhu.edu/software/hisat2/index.shtml) URL (accessed on 27 December 2023) software (version 2.0.1). The transcript abundance of individual genes was quantified by the fragment per kilobase of transcript per million reads (FPKM) method according to the length of the genes and the number of mapped read counts [27]. Differentially expressed genes (DEGs) were identified between comparison groups (API vs DIQ and SB vs DIQ) by the DESeq2 package using a false discovery rate (FDR) < 0.05 and a |log2(foldchange)| > 1 as threshold screening conditions [28]. Finally, gene ontology (GO) enrichment analysis was performed to identify cellular components, molecular functions, and biological processes of the DEGs. GO enrichment analyses were considered significantly enriched for candidate DEGs with a *p* < 0.05 via clusterProfiler (version 4.0). The Kyoto Encyclopedia of Genes and Genomes (KEGG) provides the foundational database for pathway analysis, which includes high-level functions related to biological systems and biological metabolism. The enrichment module of KOBAS (version 3.0) was used to implement the results of KEGG enrichment analysis at *p* < 0.05 [29].

### 2.7. Quantitative Real-Time PCR (qRT-PCR) Validation

qRT-PCR validated transcriptional levels of pivotal genes from samples acquired from ducks in the DIQ, API, and SB groups and verified the reliability of RNA-Seq. cDNA synthesis was performed using the PrimeScript RT Kit with gDNA Eraser (Takara, Tokyo, Japan). Subsequent qRT-PCR utilized SYBR Green PCR Master Mix (Vazyme, Nanjing, China) on a LightCycler 96 system (Roche, Basel, Switzerland) in accordance with the manufacturer’s protocols. Samples underwent pre-denaturation at 95 °C for 1 min, followed by 40 cycles of 95 °C for 10 s, 60 °C for 5 s, and 72 °C for 15 s. The primers of relevant genes (*SLC4A3*, *ADAM12*, *BAG3*, *CLDN23*, *G6PC1*, *ADAMTS4*, *HSPA8*, *TLR1-A*, *FBXO32*, and *ST13*, along with β-actin as an internal reference gene) are shown in Table 2. The 2^−∆∆Ct^ method was used to calculate.

### 2.8. Statistical Analysis

Data analysis was conducted using the Shapiro–Wilk test and Levene’s test. For the analysis of differences under the assumption of normal distribution, one-way ANOVA followed by Turkey’s post hoc test was used for multiple comparisons in SPSS software 27.0 (SPSS Inc., Chicago, IL, USA), following the preliminary arrangement of data in WPS (12.1.0.18608, Beijing, China). All data are shown as mean ± standard error, and all differences were considered significant when *p* < 0.05. Statistical visualization was implemented in GraphPad Prism v8.3 (San Diego, CA, USA).

## 3. Results

### 3.1. The Effect of Diquat on Jejunal Oxidative Stress

**Figure 1:**
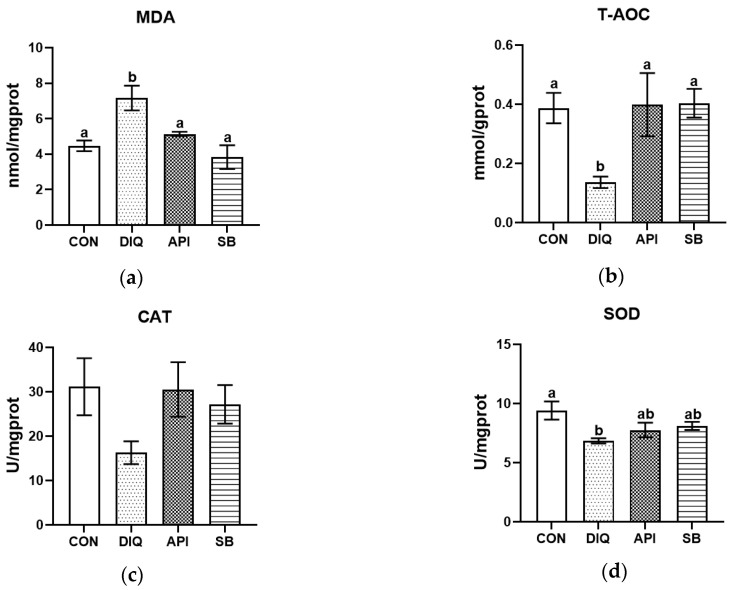
The effect of apigenin and sodium butyrate on antioxidative status activity in the jejunum of ducks induced by diquat. CON: ducks received basal diet accompanied by sterile saline injections; DIQ: ducks received basal diet accompanied by diquat injection; API: ducks received apigenin-supplemented basal diet with diquat challenge; SB: ducks received sodium butyrate-supplemented basal diet with diquat challenge. (**a**) Malondialdehyde (MDA) levels. (**b**) Total antioxidant capacity (T-AOC) levels. (**c**) Catalase (CAT) levels. (**d**) Superoxide dismutase (SOD) levels. Statistical significance assigned by letter groupings: a, b, (*p* < 0.05).

First, we determined the redox status in jejunum tissue acquired from different groups of ducks. The diquat challenge significantly increased MDA levels (*p* < 0.05) and suppressed SOD activity, as well as attenuated T-AOC when compared with the CON group (Figure 1). These changes were alleviated by dietary supplementation with apigenin and sodium butyrate, at least to a certain extent. However, no statistically significant differences were observed across the four groups in terms of the level of CAT, although the CAT levels were higher in the API and SB groups than in the DIQ group (Figure 1c). As shown in Figure 1a, MDA levels were enhanced in the diquat-treated group, but were reduced by both apigenin and sodium butyrate (*p* < 0.05). As expected, the administration of apigenin and sodium butyrate effectively prevented the reduction in T-AOC (*p* < 0.05) (Figure 1b). This inhibitory effect of diquat on SOD activity was not reversed by apigenin and sodium butyrate treatment (*p* > 0.05) (Figure 1d).

### 3.2. Jejunal Morphology

**Figure 2:**
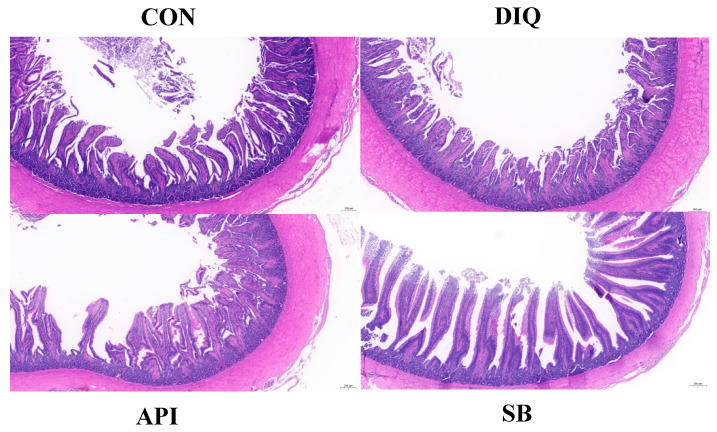
The jejunal morphological appearance of ducks in different groups. CON: ducks received basal diet accompanied by sterile saline injections; DIQ: ducks received basal diet accompanied by diquat injection; API: ducks received apigenin-supplemented basal diet with diquat challenge; SB: ducks received sodium butyrate-supplemented basal diet with diquat challenge.

The jejunal morphology of ducks in different groups is shown in Figure 2. The diquat injection significantly reduced villus height (*p* < 0.05) (Table 3). The villus height: crypt depth ratio in the DIQ group tended to be reduced compared with the CON group. Interestingly, sodium butyrate treatment partially alleviated histological morphological injury in the jejunum of ducks in the SB group (Table 3). Sodium butyrate significantly enhanced jejunal villus height and villus height: crypt depth ratio in oxidatively stressed ducks relative to the DIQ group (*p* < 0.05) (Table 3). No statistically significant difference was observed between the API and DIQ groups in this respect (*p* > 0.05) (Table 3).

### 3.3. Overview of Sequence Reads

To detect the effects of apigenin and sodium butyrate on the jejunum of ducks under oxidative stress, we generated RNA-seq data from ducks in the DIQ, API, and SB groups. The average number of raw reads obtained for each sample was 43,811,989, with an average of 42,618,060 clean reads from each sample after trimming for low quality and the removal of adapters. The clean reads ratio did not fall below 96.46%. In the jejunum of each sample from the three groups, the Q20 value and Q30 values exceeded 97.56% and 93.21%, respectively. The average number of mapped reads collected from each sample was 37,236,235, whereas the mapping rate of clean reads mapped to reference genes ranged from 85.27% to 88.78% (Table 4).

### 3.4. Differential Gene Expression Analysis

**Figure 3:**
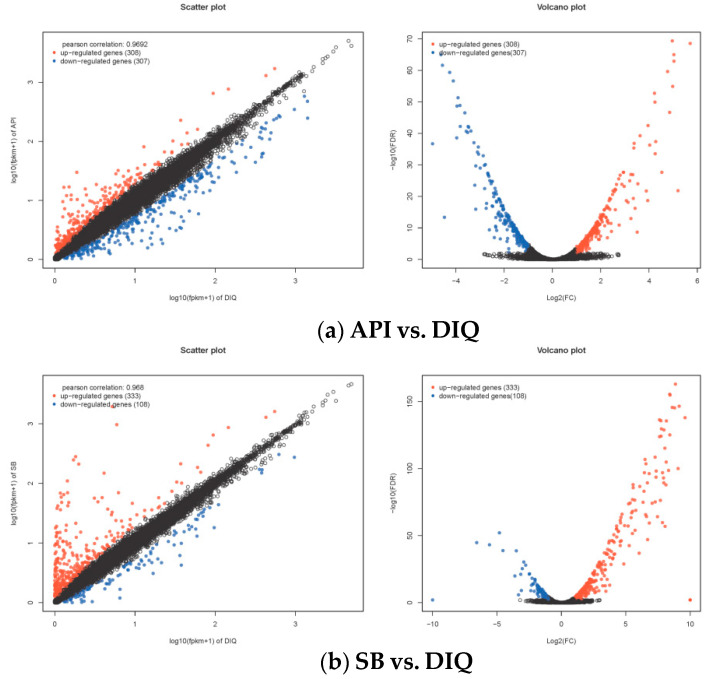
The scatter plot and volcano plot of differentially expressed mRNAs between the different comparisons. (**a**) The scatter plot and volcano plot between API and DIQ. (**b**) The scatter plot and volcano plot between SB and DIQ. DIQ: ducks received basal diet accompanied by diquat injection; API: ducks received apigenin-supplemented basal diet with diquat challenge; SB: ducks were fed the basal diet containing sodium butyrate with diquat injection. Red: up-regulated genes; Blue: down-regulated genes; Black: non-significant genes.

**Figure 4:**
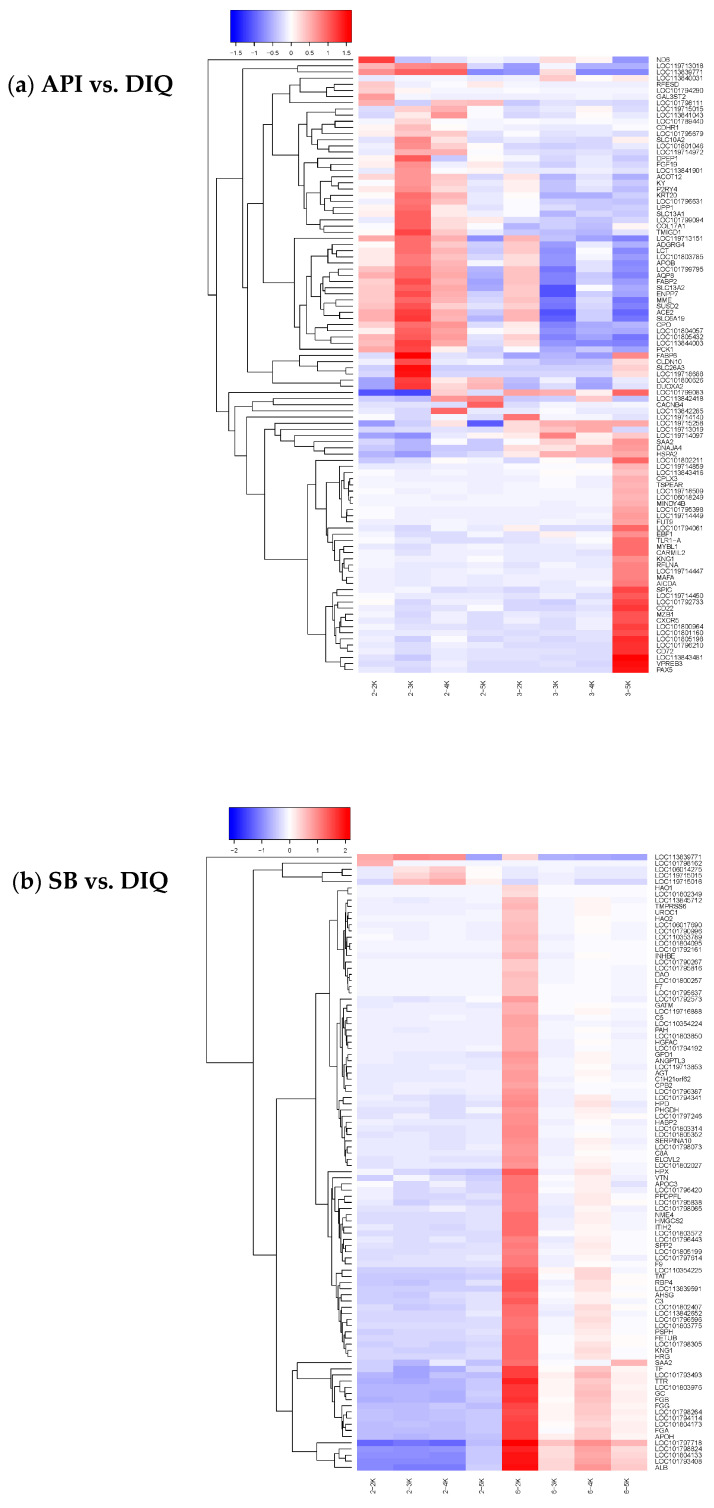
Hierarchical clustering plots of differentially expressed mRNAs between the different comparisons. (**a**) Hierarchical clustering plots of between API (3-2K, 3-3K, 3-4K, and 3-5K) and DIQ (2-2K, 2-3K, 2-4K, and 2-5K). (**b**) Hierarchical clustering plots of between SB (6-2K, 6-3K, 6-4K, and 6-5K) and DIQ (2-2K, 2-3K, 2-4K, and 2-5K). DIQ: ducks received basal diet accompanied by diquat injection; API: ducks received apigenin-supplemented basal diet with diquat challenge; SB: ducks were fed the basal diet containing sodium butyrate with diquat injection.

Next, we investigated differences in gene expression for two comparisons (API vs DIQ and SB vs DIQ). Specifically, RNA-seq analysis revealed 615 differentially expressed genes (DEGs) in API versus DIQ comparison, of which 308 genes were expressed at markedly higher levels in the API group and 307 genes in the DIQ group (Figure 3a). In total, comparative analysis detected 441 DEGs with significant expression divergence between the SB and DIQ groups (333 upregulated and 108 downregulated) (Figure 3b). To identify DEGs in the jejunums for different comparisons, we performed hierarchical cluster analysis. As shown in Figure 4, ducks from the DIQ, API, and SB groups were clustered together.

### 3.5. Functional Enrichment Analysis

**Figure 5:**
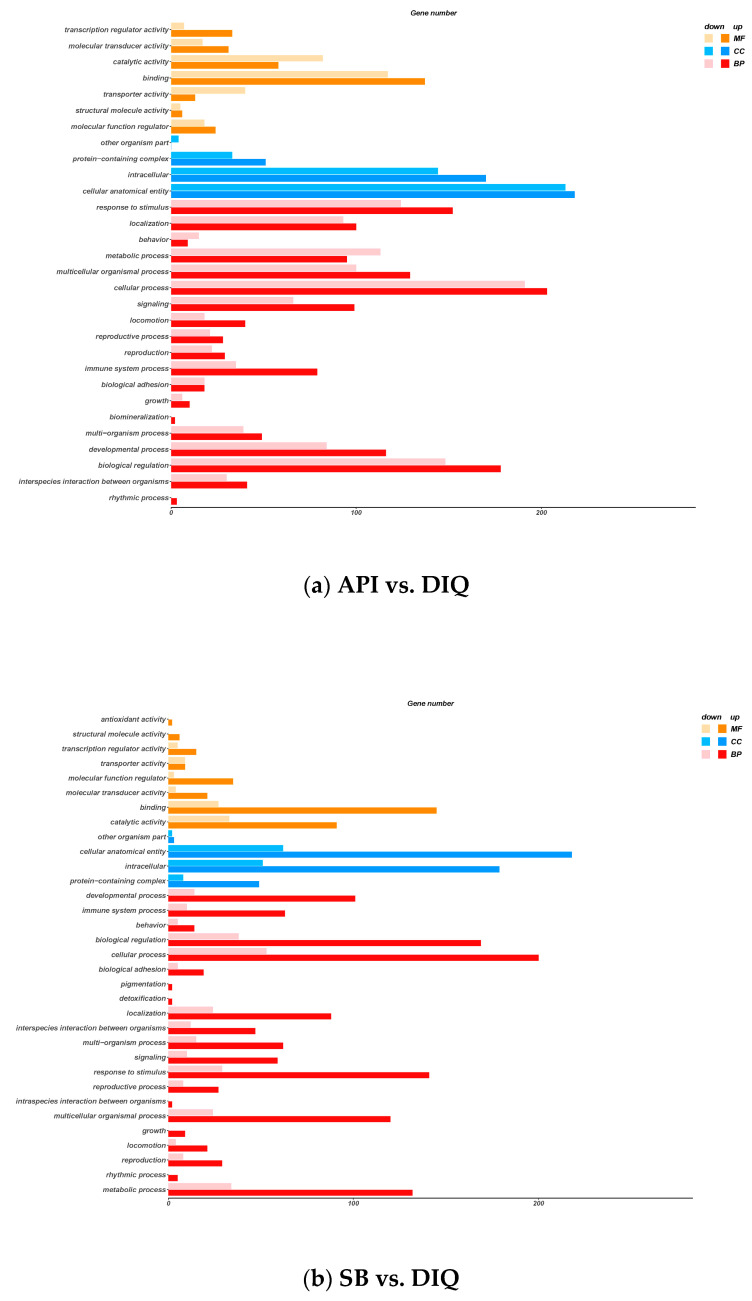
Gene ontology (GO) enrichment analysis. (**a**) GO enrichment analysis between the API and DIQ groups. (**b**) GO enrichment analysis between the SB and DIQ groups. The X-axis represents the significance enrichment number for genes, and the Y-axis represents the GO term.

To comprehensively characterize the biological functions of the 615 DEGs from the API vs DIQ comparison and the 441 DEGs from the SB vs DIQ comparison, we next performed GO enrichment analysis to search for significantly over-represented categories. In general, the DEGs were classified into three GO categories: molecular function (MF), cellular components (CC), and biological process (BP). Results arising from the comparison between API and DIQ are presented in Figure 5a. GO term analysis for jejunum tissues from the API and DIQ groups revealed various molecular functions (MFs), including transcription’s regular activity, molecular transducer activity, and catalytic activity. This analysis identified cellular components (CCs) related to protein-containing complex, intracellular, and cellular anatomical entities. The analysis of biological processes (BPs) revealed the regulation of response to stimulus, cellular processes, and metabolic processes. The results arising from GO enrichment analysis between the SB and DIQ groups are displayed in Figure 5b; the annotated results of MF were mainly related to antioxidant activity, binding activity, and catalytic activity. In the CC category, protein-containing complex, intracellular, and cellular anatomical entities were annotated. The developmental process, biological regulation, and cellular processes were enriched in the BP category.

**Figure 6:**
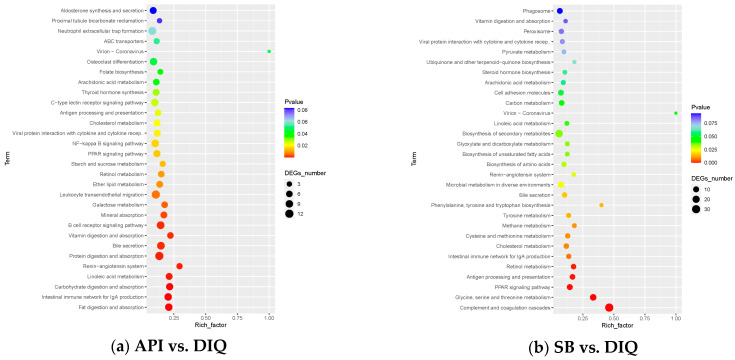
Kyoto Encyclopedia of Genes and Genomes (KEGG) enrichment analysis. (**a**) KEGG enrichment analysis between the API and DIQ groups. (**b**) KEGG enrichment analysis between the SB and DIQ groups.

Subsequently, all DEGs were integrated into the KEGG pathway database. The KEGG enrichment analysis of DEGs from the API vs DIQ comparison is shown in Figure 6a. Enrichment was observed for the PPAR signaling pathway, the NF-ĸB signaling pathway, the B cell receptor signaling pathway, the intestinal immune network for IgA production, protein digestion and absorption, and vitamin digestion and absorption. In addition, DEGs from the SB vs DIQ comparison were highly enriched in the PPAR signaling pathway, the NF-ĸB signaling pathway, cell adhesion molecules, and microbial metabolism in diverse environments (Figure 6b).

### 3.6. qRT-PCR Validation

**Figure 7:**
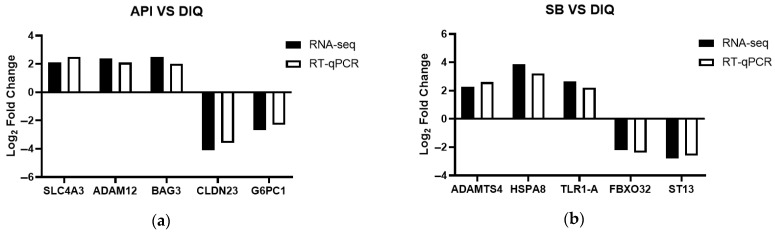
RNA-seq Validation (qPCR). (**a**) Histogram of RNA-seq and qRT-PCR expression levels for specific genes in the API and DIQ groups. (**b**) Histogram of RNA-seq and qRT-PCR expression levels for specific genes in the SB and DIQ groups. *SLC4A3*, solute carrier family 4 member 3; *ADAM12*, ADAM metallopeptidase domain 12; *BAG3*, B-cell lymphoma 2-associated-athanogene 3; *CLDN23*, claudin 23; *G6PC1*, glucose-6-phosphatase catalytic subunit 1; *ADAMTS4*, ADAM metallopeptidase with thrombospondin type 1 motif 4; *HSPA8*, heat shock protein family A (Hsp70) member 8; *TLR1-A*, toll-like receptor 1; *FBXO32*, F-box protein 32; *ST13*, suppression of tumorigenicity 13.

To validate the RNA-seq data fidelity, we randomly selected target genes (*SLC4A3*, *ADAM12*, *BAG3*, *CLDN23*, *G6PC1*, *ADAMTS4*, *HSPA8*, *TLR1-A*, *FBXO32*, and *ST13*) from the DIQ, API, and SB groups for qRT-PCR analysis. As shown in Figure 7, the qRT-PCR revealed expression patterns for these target genes that were consistent with those of the RNA-seq analysis, thus indicating the reliability of the RNA-seq data.

## 4. Discussion

The small intestine mediates critical nutrient digestion, absorption, and homeostatic regulation. The intestinal epithelial barrier provides an obstruction that separates hazardous inducers and pathogenic microorganisms, representing the first line to separate the intestinal lumen from the external environment [30]. Consequently, the small intestine is highly susceptible to oxidative stress induced by moldy feed and residual medicines. Diquat, as an inducer of oxidative stress, is commonly used to generate in vivo models of oxidative stress by injection, thus allowing the investigation of the effects of oxidative stress on organs, including the intestine, liver, kidney, and lung injury [31,32,33]. 

According to our results, diquat treatment significantly increased MDA levels but reduced the activities of T-AOC and SOD in the jejunum of ducks in the DIQ group when compared with those in the CON group, thus indicating that diquat injection caused oxidative stress by impairing the antioxidant indices of the jejunum. Similarly, the levels of MDA were markedly elevated, while the levels of SOD and T-AOC were significantly reduced in the liver and serum of broilers following diquat treatment compared to a control group [9]. Collectively, these results are partially consistent with our present results, except for differences in CAT levels, which are probably due to the different breeds and numbers of animals, along with the timing and injection of diquat. 

Apigenin, a dietary flavonoid, occurs naturally in various fruits and vegetables, especially celery, and exhibits excellent antioxidant, anti-inflammatory, and anti-tumor properties [34,35]. Recent research on apigenin has provided evidence that it is able to relieve oxidative stress in pullets by regulating antioxidant indicators, including SOD, T-AOC, and MDA [36]. Studies relating to the antioxidant effects of apigenin have predominantly focused on cellular experiments, with very few experimental studies performed in vivo. A previous study provided reliable evidence that apigenin reduces oxidative stress and inhibits the MAPK/NF-κB pathway to inhibit pyroptosis [37]. In addition, apigenin can improve the autophagy of cells suffering oxidative stress damage by inhibiting the PI3K/Akt/mTOR pathway [38]. Sodium butyrate, a type of natural butyrate acid, is produced by intestinal microorganisms via the fermentation of dietary fibers and carbohydrates [39]. To some extent, sodium butyrate is now considered a food additive to maintain the integrity of the intestinal mucosa, provide energy for epithelial cells, and exert potent anti-oxidative effects in animals [40,41]. In addition, several studies have demonstrated that sodium butyrate contributes to intestinal metabolism, suppresses inflammation, and improves intestinal barrier function [20,42,43]; these results are similar to our present findings. In the present study, sodium butyrate treatment partly alleviated oxidative stress by up-regulating the levels of T-AOC while down-regulating the levels of MDA. In a previous study, analysis revealed a marked elevation in MDA levels and a reduction in SOD2 levels in the serum of LPS-treated rats when compared with control rats [44]. The present findings support the hypothesis that the addition of apigenin and sodium butyrate relieves oxidative stress in the jejunum of diquat-treated ducks by regulating related antioxidant factors.

It is generally known that intestinal epithelial integrity depends critically on mucosal architecture as the principal barrier to luminal aggressors, which dictates barrier functional competence. Previous studies have demonstrated that diquat causes oxidative stress and damages intestinal morphology, including the intestinal villus height, crypt depth, and villus height/crypt depth, which are key morphological indicators that reflect intestinal integrity and permeability as well as its absorptive capacity [45,46]. Exposure to diquat is known to reduce villus height in the intestinal structure of piglets; these findings concur with our present results [47]. Conversely, we found that sodium butyrate supplementation ameliorated these phenomena. According to the results of our study, ducks under oxidative stress and fed sodium butyrate demonstrated statistically elevated jejunal villus height and villus height/crypt depth when compared to ducks from the DIQ group. Similarly, as sodium butyrate supplementation increased, the villus height in the jejunum increased, although crypt depth did not change markedly [48]. Previous studies have shown that sodium butyrate supplementation significantly enhances jejunal villus height in broilers versus controls [49]. Little is known about the effects of apigenin on intestinal morphology, except for our published manuscripts [36]. The present work establishes that apigenin was not as effective as sodium butyrate in alleviating the damage induced by diquat on the intestinal morphology. 

To elucidate the specific mechanisms responsible for the beneficial effects of apigenin and sodium butyrate on jejunal oxidative injury in ducks, we further assessed the effects of apigenin and sodium butyrate on the transcriptome of the jejunum in ducks suffering from oxidative stress induced by diquat. KEGG analysis revealed that the DEGs identified between the API and DIQ groups were mainly distributed in the PPAR signaling pathway, the NF-ĸB signaling pathway, the B cell receptor signaling pathway, the intestinal immune network for IgA production, protein digestion and absorption, and vitamin digestion and absorption. The DEGs between the SB and DIQ groups were enriched in the PPAR signaling pathway, the NF-ĸB signaling pathway, cell adhesion molecules, and microbial metabolism in diverse environments. According to our previous results, apigenin acts as a modulator of PPAR-γ and can ameliorate non-alcoholic fatty liver disease via the novel regulation of PPAR-γ and the inhibition of oxidative stress [50]. Sodium butyrate has been reported to activate the PPAR pathway to protect against hepatocyte lipoatrophy injury [51]. A previous study demonstrated that sodium butyrate plays an anti-inflammatory role by regulating NF-κB transcriptional activity [52]. High glucose-induced inflammation was inhibited by sodium butyrate to control the acetylation of NF-κB p65 in human monocytes [53]. Our sequencing results concur with those derived from previous studies. Pathway gene analysis from the jejunum of ducks following apigenin and sodium butyrate supplementation with diquat injection revealed the modulation of many genes, including *SLC4A3*, *ADAM12*, *BAG3*, *CLDN23*, *G6PC1*, *ADAMTS4*, *HSPA8*, *TLR1-A*, *FBXO32*, and *ST13*. *SLC4A3* (solute carrier family 4 member 3) belongs to the solute carrier (SLC) superfamily, which is the largest transporter family and is mainly situated on cellular and organelle membranes [54]. *BAG3* (B-cell lymphoma 2-associated-athanogene 3) is responsible for the quality control of misfolded proteins by interaction with the ATPase domain of *HSP70* [55]. Previous research reported that the overexpression of BAG3 alleviated damage induced by ischemic stroke by activating autophagy and inhibiting apoptosis [56]. HSPA8 (heat shock protein family A (*Hsp70*) member 8), which is a member of the *HSP70* family, mediates protein assembly, refolding, and degradation to regulate cellular proteostasis [57]. Prior studies elucidated *HSPA8*’s novel mechanistic role in immune function, cellular senescence, and apoptotic pathways [58]. Overall, our study provides a wider understanding of the beneficial mechanisms of apigenin and sodium butyrate on oxidative stress in the jejunum of ducks induced by diquat; however, the validation of target proteins and associated pathways needs to be investigated further in the future.

## 5. Conclusions

The dietary supplementation of apigenin and sodium butyrate alleviated intestinal oxidative stress by modulating the levels of antioxidant markers and mRNA expression of jejunum tissues at the transcriptome level in diquat-induced ducks. However, the functional validation constraints of gene expression findings underscore the need for in-depth studies to uncover the precise molecular mechanisms driving these biological phenomena. Future research will focus on protein-level investigations of candidate genes. Collectively, our findings provide new insight into the utility of apigenin and sodium butyrate as promising antioxidant additives.

## Figures and Tables

**Table 1:** Composition and nutritional profile of the basal diet.

Composition	Content (%)	Nutrient Levels ^2^	Content
Corn	46.5	Metabolic energy (MJ/kg)	11.06
Soybean meal	26.6	Crude protein, %	17.85
Wheat bran	2.1	Lysine, %	1.1
Rice bran	15.6	Cysteine + Methionine, %	0.68
Limeston	6.3	Calcium, %	2.67
NaCl	0.4	Available phosphorus, %	0.37
Premix ^1^	2.5		
Total	100		

^1^ The premix provided the following nutrients per kilogram of diet: VA, 12,300 IU; VE, 20 IU; VK3, 2.73 mg; VB1, 2.00 mg; VB2, 6.00 mg; VB6, 3.00 mg; Folic acid, 12.00 mg; Pantothenic acid, 50.00 mg; Niacin, 50.00 mg; Cu, 6 mg; Fe, 80 mg; Zn, 40 mg; Mn, 100 mg; Se, 0.15 mg; I, 0.35 mg. ^2^ Nutrient levels was a calculated value.

**Table 2:** Primers for Quantitative Real-Time PCR analysis.

Genes	Primer (From 5′ to 3′)	Amplicon Length (bp)	GenBank Number
*SLC4A3*	F: CCCTTTGAAGCGGACTGGAATAT	163	XM_021277160.3
	R: CCCGAAGGTGATGGCAGGA		
*ADAM12*	F: ACAAGGCAAGGATGTGGAAA	166	XM_038181074.1
	R: ATGGAGGCTGGTGAAAGGAT		
*BAG3*	F: CGGCAAACGGTCCGTCTCGT	194	XM_027460145.2
	R: GGTGGGCACAGCCTCTGTCTT		
*CLDN23*	F: TGGAGGACGAGCGAGACGGG	156	XM_021272456.3
	R: GAGATTCAGGCTGGGTCCTTGTT		
*G6PC1*	F: GCCATCCAGCAGTTCCCACTCAC	181	XM_027445511.2
	R: AGAAGCCCGTCCAAAGCACCAG		
*ADAMTS4*	F: GCGCCCGCTTCATCACCGATTTCC	155	XM_038172665.1
	R: GTGCCGCGAGTCCATGCCGAAA		
*HSPA8*	F: CAGCCTATTTCAACGACTCCCA	140	XM_027444224.2
	R: CCTTTCAGCACCGACCTTCTT		
*TLR1-A*	F: TCTTTCTACTTGCTGGCACA	121	XR_005265145.1
	R: AAGGCTTCGGCATACTCA		
*FBXO32*	F: TGCTGGAGCTGATAGCGAAGT	189	HM627858.1
	R: CAGATTTGCCGACCCGTTG		
*ST13*	F: TGAACTTCAGAAGGCTGTCGA	176	XM_027451163.2
	R: ATTTGTAGGTCTGTGCCGAGT		
*β-actin*	F: ATGTCGCCCTGGATTTCG	135	EF667345.1
	R: CACAGGACTCCATACCCAAGAAT		

**Table 3:** Effects of apigenin and sodium butyrate on jejunal morphology.

Items	CON	DIQ	API	SB
Villus height (μm)	688.66 ^a^ ±36.22	414.43 ^b^ ± 36.80	473.10 ^b^ ± 6.256	723.63 ^a^ ± 30.73
Crypt depth (μm)	285.74 ^a^ ± 23.76	219.03 ^ab^ ± 10.95	206.07 ^b^ ± 13.69	199.27 ^b^ ± 31.73
Villus height/crypt depth	2.49 ^b^ ± 0.27	1.89 ^b^ ± 0.09	2.32 ^b^ ± 0.18	3.63 ^a^ ± 0.58

Note: means in the same row, not sharing superscript letters, are significantly different (*p* < 0.05). CON: ducks received basal diet accompanied by sterile saline injections; DIQ: ducks received basal diet accompanied by diquat injection; API: ducks received apigenin-supplemented basal diet with diquat challenge; SB: ducks received sodium butyrate-supplemented basal diet with diquat challenge.

**Table 4:** Sequence quality and alignment information in three groups of ducks.

Terms	Raw Reads	Clean Reads	Clean Reads Ratio	Q20	Q30	Mapped Reads	Mapping Ratio
2-2K	41,791,188	40,804,162	97.64%	97.98%	94.32%	35,503,701	87.01%
2-3K	43,848,638	42,669,742	97.31%	97.83%	93.92%	37,882,196	88.78%
2-4K	46,330,764	45,146,352	97.44%	98.13%	94.73%	38,496,294	85.27%
2-5K	41,404,312	40,401,026	97.58%	98.11%	94.71%	35,104,451	86.89%
3-2K	47,602,426	46,390,366	97.45%	97.84%	93.92%	40,076,637	86.39%
3-3K	42,445,702	41,338,746	97.39%	98.15%	94.9%	36,034,984	87.17%
3-4K	45,998,644	44,875,454	97.56%	97.88%	94.1%	39,001,257	86.91%
3-5K	41,093,628	39,637,998	96.46%	97.72%	93.66%	35,071,700	88.48%
6-2K	41,361,382	40,267,496	97.36%	98.19%	94.94%	35,487,744	88.13%
6-3K	45,781,654	44,301,954	96.77%	97.77%	93.75%	38,910,406	87.83%
6-4K	42,965,146	41,579,996	96.78%	97.56%	93.21%	36,507,236	87.80%
6-5K	45,120,390	44,003,424	97.52%	97.87%	94.01%	38,758,215	88.08%

## Data Availability

The raw sequence data were deposited in the National Genomics Data Center under the accession number GSA: CRA025140 (https://ngdc.cncb.ac.cn/gsa) URL (accessed on 1 May 2025).

## Abbreviations

The following abbreviations are used in this manuscript:

SOD	Superoxide dismutase
MDA	Malondialdehyde
CAT	Catalase
T-AOC	Total antioxidant capacity
SLC4A3	Solute carrier family 4 member 3
ADAM12	ADAM metallopeptidase domain 12
BAG3	B-cell lymphoma 2-associated-athanogene 3
CLDN23	Claudin 23
G6PC1	Glucose-6-phosphatase catalytic subunit 1
ADAMTS4	ADAM metallopeptidase with thrombospondin type 1 motif 4
HSPA8	Heat shock protein family A (Hsp70) member 8
TLR1-A	Toll-like receptor 1
FBXO32	F-box protein 32
ST13	Suppression of tumorigenicity 13
